# Differences in Fruit and Vegetable Intake by Race/Ethnicity and by Hispanic Origin and Nativity Among Women in the Special Supplemental Nutrition Program for Women, Infants, and Children, 2015

**DOI:** 10.5888/pcd13.160130

**Published:** 2016-08-25

**Authors:** Jennifer Di Noia, Dorothy Monica, Karen Weber Cullen, Rafael Pérez-Escamilla, Heewon Lee Gray, Alla Sikorskii

**Affiliations:** Author Affiliations: Dorothy Monica, Saint Joseph’s WIC Program, Paterson, New Jersey; Karen Weber Cullen, USDA/ARS Children’s Nutrition Research Center, Baylor College of Medicine, Houston, Texas; Rafael Pérez-Escamilla, Yale School of Public Health, New Haven, Connecticut; Heewon Lee Gray, Teachers College, Columbia University, New York, New York; Alla Sikorskii, Michigan State University, East Lansing, Michigan.

## Abstract

**Introduction:**

The objective of this exploratory study was to determine whether fruit and vegetable consumption differed by race/ethnicity, by origin and nativity among Hispanics, and by language preference (as an indicator of acculturation) among foreign-born Hispanics.

**Methods:**

We recruited 723 women enrolled in the Special Supplemental Nutrition Program for Women, Infants and Children (WIC) and orally administered a questionnaire containing demographic items, validated measures of food security status and social desirability trait, and the Behavioral Risk Factor Surveillance System fruit and vegetable module. Differences in intakes of 100% fruit juice, fruit, cooked or canned beans, and dark green, orange-colored, and other vegetables were assessed by using analysis of covariance with Bonferroni post hoc tests. Analyses were controlled for age, pregnancy status, breastfeeding status, food security status, educational attainment, and social desirability trait.

**Results:**

The frequency of vegetable intake differed by race/ethnicity (cooked or canned beans were consumed more often among Hispanic than non-Hispanic black and non-Hispanic white or other participants, orange-colored vegetables were consumed more often among Hispanics than non-Hispanic black participants, and other vegetables were consumed more often among non-Hispanic white or other than among non-Hispanic black and Hispanic participants), origin (other vegetables were consumed more often among Columbian and other Hispanics than Dominican participants) and nativity (orange-colored vegetables were consumed more often among foreign-born than US-born Hispanics). Fruit and vegetable intake did not differ by language preference among foreign-born Hispanics.

**Conclusion:**

Differences in fruit and vegetable consumption among WIC participants by race/ethnicity and by Hispanic origin and nativity may have implications for WIC nutrition policies and nutrition education efforts.

## Introduction

The US population does not meet dietary guidelines (1) for intake of vegetables in any vegetable subgroup (dark green, red and orange, legumes, starchy, or other), and most (80%) do not meet recommendations for intake of fruit (whole fruit or 100% juice). Low-income populations are among those least likely to meet guidelines (2). A better understanding of fruit and vegetable intake in this population is needed, in particular, fruits and vegetables emphasized in dietary guidelines.

Differences by race/ethnicity are found in total intake of fruit, fruit juice, legumes, plantains, and root crops (higher among Hispanics than non-Hispanic whites), total vegetables, potatoes, other vegetables, and green salad (higher among non-Hispanic whites than Hispanics), and non-citrus fruit (higher among Hispanic than non-Hispanic black and and non-Hispanic white women) and by nativity in intakes of fruits and legumes (consumed by more foreign-born than US-born Hispanics) (3–6). As acculturation increases among foreign-born Hispanics, intake of fruit, beans, and starchy root vegetables decreases (7,8). Differences by origin are found in intake of white potatoes. South American women eat more white potatoes than do Mexican, Central American, Caribbean, and Spanish women and women of multiple Hispanic origin and more green salad than Central American women. Mexican, Central American, Caribbean, and Spanish women eat more cooked dried beans than do South American women (9,10). Whether differences by race/ethnicity occur among low-income women and by origin and nativity among low-income Hispanic women is unclear. The objective of this exploratory study was to determine whether intake of fruits and vegetables differed by race/ethnicity, origin, and nativity among Hispanics, and by language preference among foreign-born Hispanics (as an indicator of acculturation). 

## Methods

We conducted an exploratory study of 723 low-income women enrolled in the Special Supplemental Nutrition Program for Women, Infants, and Children (WIC). Of these, 436 were Hispanic, and of these, 244 were foreign-born. Information on foods made available to women in the WIC program is available elsewhere (11).

### Design and sample

This cross-sectional study examined baseline data from WIC Fresh Start, a randomized controlled trial of nutrition education to promote purchases and consumption of fruits and vegetables sold at farmers’ markets among women enrolled in WIC. The trial is described elsewhere (12). Eligibility criteria for the trial were being pregnant, postpartum, or a female caregiver of an infant or child enrolled in WIC; having no known dietary restrictions; and not being at high risk for nutritional or health problems (as defined by WIC). Participants were recruited from among women in the waiting room of a large WIC agency located in a densely populated urban area of New Jersey by research assistants fluent in both English and Spanish. Of the 1,345 women approached, 64 did not meet eligibility criteria, 537 were eligible but declined to participate, and 744 were enrolled (58% consent rate). This study examined data provided by WIC Fresh Start participants reporting one race/ethnicity (N = 723). Participants provided informed written consent. The study was approved by the William Paterson University Institutional Review Board for Human Subject Research (no. 2014–368). Data were collected from June 1, 2015, through August 12, 2015. 

### Measurement and statistical analyses

We orally administered a questionnaire that consisted of closed-ended, multiple-choice questions assessing participants’ race/ethnicity, nativity (US-born, born outside of the United States [defined as outside of the United States and its territories, including Puerto Rico]) and language preference (English, Spanish, other, and, if “other,” what that preferred language was). We made Hispanic/Latina a choice of race because many Hispanic WIC participants consider their ethnicity their race (13). After reporting race/ethnicity, participants were asked about their origin (eg, “If you answered Hispanic/Latina [to the question on race/ethnicity], what is your origin or origins, for example Puerto Rican, Mexican, Dominican, Columbian and so on?”). For those reporting more than one origin (40 [6%]), origin was coded as the first origin reported. Some researchers advocate grouping Hispanic origin countries by geographic region (eg, grouping Mexican, Mexican American, and Chicano together as Mexican) because geographic regions tend to share sociopolitical history and culture (9). However, health needs and health outcomes differ significantly among Hispanics by country of origin (14). For this reason, we did not group Hispanic participants by region in this study so as not to obscure differences in fruit and vegetable intake among Hispanics from different countries within the same region. Previous work has shown that greater acculturation is associated with English language preference than with Spanish language preference (15). For this reason, foreign-born Hispanics who preferred to speak English were considered more acculturated than were those who preferred to speak Spanish.

Participants reported their birthdate (used to calculate their age in years), pregnancy status, breastfeeding status, and educational attainment and completed validated measures of food security status and social desirability trait (a response set reflecting the tendency to respond in a manner consistent with perceived social norms) (16,17). Participants were classified as food secure or insecure on the basis of their response to the food security measure. On the measure of social desirability trait, scores ranged from 0 to 10, with higher scores indicating a higher social desirability trait. Analyses were adjusted for the potentially biasing effects of these variables on fruit and vegetable intake.

We used the 2013 Behavioral Risk Factor Surveillance System (BRFSS) fruit and vegetable module to measure intake of 100% fruit juice, fruit, cooked or canned beans, and dark green, orange-colored, and other vegetables (18). The instrument has moderate validity (correlations with diet records, 24-hour recalls, and food frequency questionnaires range from 0.29 to 0.63) and reliability (test–retest correlations and κ values range from 0.19 to 0.77) (19). Respondents could report their intake as the number of times per day, week, or month the included foods were consumed. For the WIC Fresh Start trial, we changed the reference period from the previous month to the previous 2 weeks. Before presenting the questions, the interviewer read the following: “These next questions are about the fruit and vegetables you ate or drank during the past 2 weeks.” The original response categories were retained. As such, participants could report their intake as times per month foods were consumed because it was assumed that intake occurred uniformly (ie, a response of “2 times a month” was equivalent to a response of “once every 2 weeks” ). Interviewers followed BRFSS guidelines on which foods and juices to count in fruit and vegetable categories (Appendix).

We examined BRFSS fruit and vegetable item response distributions, and all but one (the distribution for the item assessing fruit intake) were positively skewed. Square-root transformations were therefore applied to improve the normality of the distributions (transformed data are presented). Descriptive statistics were used to characterize the sample. Differences in fruit and vegetable intake by race/ethnicity were examined with analysis of covariance (ANCOVA) with Bonferroni post hoc tests adjusted for covariates. Subgroup analyses among Hispanic participants (N = 436) examined differences in intake by origin and nativity. A final set of ANCOVAs conducted among foreign-born participants (N = 244) examined whether consumption differed by language preference. To gauge the practical significance of differences found (in addition to the statistical significance of the differences), effect sizes were computed. Effect sizes were expressed as Cohen *d*, the difference between subgroup means divided by the adjusted standard deviation (square root of the mean-squared error). A Cohen *d* of 0.20 is considered small, 0.50 is considered medium, and 0.80 is considered large (20). There was 80% power to detect medium effect sizes in tests of intake differences by race/ethnicity and nativity and large effect sizes in tests of intake differences by Hispanic origin and language preference. Analyses were conducted in 2016 with SPSS for Windows, version 23, 2015 (IBM Inc). Across analyses, we used a *P* value of .05 to establish significance.

## Results

The sample of 723 participants had a mean age of 29.0 years (standard deviation [SD] 6.9 y); 17% were pregnant, 22% were breastfeeding, 55% were food insecure, 60% were Hispanic, 31% were non-Hispanic black, and 9% were non-Hispanic white or other. The largest origin groups among Hispanics were Dominican (n = 159, 36%), Puerto Rican (n = 103, 24%), Mexican (n = 43, 10%), Peruvian (n = 33, 8%), and Columbian (n = 28, 6%). Nearly three-fifths (56%) of Hispanics were born outside the United States. Although 229 (53%) Hispanics preferred to speak English, most foreign-born Hispanics (n = 244) preferred to speak Spanish (n = 190, 78%). Fifty percent of the sample reported a high school or general equivalency degree or less. The mean score in the sample on the measure of social desirability trait was 7.7 (SD, 1.7) (Table 1). Relative to WIC Fresh Start participants, higher percentages of women served by the collaborating WIC agency were pregnant (44%) and breastfeeding (36%). Sixty-six percent of the WIC clinic population was Hispanic, 13% was non-Hispanic black, and 21% was non-Hispanic white or other.

**Table 1:** Characteristics of Participants (N = 723) in Study of Differences in Intake of Fruits and Vegetables by Race/Ethnicity and by Hispanic Origin and Nativity Among Women in the Special Supplemental Nutrition Program for Women, Infants, and Children (WIC), 2015

Characteristic	Number (%)^a^
**All Participants**	
**Age, mean (SD), y**	29.0 (6.9)
**Pregnant**	124 (17)
**Breastfeeding**	157 (22)
**Food insecure**	396 (55)
**Social desirability trait, mean (SD)^b^**	7.7 (1.7)
**Race/ethnicity**	
Non-Hispanic black	221 (31)
Hispanic/Latina	436 (60)
Non-Hispanic white or other	66 (9)
**Nativity**	
United States	431 (60)
Outside United States	292 (40)
**Educational attainment**	
Elementary school (grades 1–8)	27 (4)
Some high school (grades 9–12), no diploma	103 (14)
High school or General Equivalency Degree or equivalent	235 (32)
More than high school	358 (50)
**Non-Hispanic Race/Ethnicity**	
**Origin**	
**Non-Hispanic black^c^**	
Jamaican	18 (52)
Nigerian	4 (11)
Other origin	13 (37)
**Non-Hispanic white or other^c^**	
Italian	18 (41)
Irish	9 (21)
Other origin	17 (38)
**Nativity**	
**Non-Hispanic black**	
United States	188 (85)
Outside United States	33 (15)
**Non-Hispanic white or other**	
United States	51 (77)
Outside United States	15 (23)
**Hispanic Race/Ethnicity^d^**	
**Origin**	
Dominican	159 (36)
Puerto Rican	103 (24)
Mexican	43 (10)
Peruvian	33 (8)
Columbian	28 (6)
Other Hispanic origin	70 (16)
**Nativity**	
United States	192 (44)
Outside United States	244 (56)
**Preferred language**	
English	229 (53)
Spanish	207 (47)
**Language preference by nativity**	
**United States**	
English language preference	175 (91)
Spanish language preference	17 (9)
**Outside United States**	
English language preference	54 (22)
Spanish language preference	190 (78)

a Values are n (%) unless otherwise indicated.

b The tendency to respond in a manner consistent with perceived social norms. Measured with a short form of the Marlowe-Crowne Social Desirability Scale (M-C 2[10]) (16); scores ranged from 0 to 10, with higher scores indicating a higher social desirability trait. Sample size = 719.

c Thirty-five non-Hispanic black and 44 non-Hispanic white participants reported an origin.

d Hispanic/Latina was offered as a choice of race in the study questionnaire because many Hispanic WIC participants consider their ethnicity their race (13).

We found a significant association between race/ethnicity and intake of cooked or canned beans, orange-colored vegetables, and other vegetables (Table 2). Hispanic participants had a higher frequency of intake of cooked or canned beans than non-Hispanic blacks (adjusted mean difference, 0.16; 95% confidence interval [CI], 0.09–0.23]; Cohen *d* = 0.45, *P* < .001) and non-Hispanic white or other participants (adjusted mean difference, 0.18; 95% CI, 0.06–0.29; Cohen *d* = 0.51, *P* = .001) and a higher frequency of intake of orange-colored vegetables than non-Hispanic blacks (adjusted mean difference, 0.14; 95% CI, 0.07–0.21; Cohen *d* = 0.41; *P* < .001). Non-Hispanic white or other participants had a higher frequency of intake of other vegetables than did non-Hispanic blacks (adjusted mean difference, 0.16; 95% CI, 0.02–0.30; Cohen *d* = 0.41; *P* = .01) and Hispanics (adjusted mean difference, 0.16; 95% CI, –0.29 to –0.03; Cohen *d* = 0.41; *P* = .008).

**Table 2:** Differences in Intake of Fruits and Vegetables by Race/Ethnicity Among Women in the Special Supplemental Nutrition Program for Women, Infants, and Children, 2015

Food	Adjusted Mean Difference (95% Confidence Interval)^a^^,^^b^		
	Hispanic (N = 221)	Non-Hispanic Black (N = 221)	Non-Hispanic White or Other (N = 66)
100% fruit juice	Reference	−0.02 (−0.13 to 0.10)	0.01 (−0.18 to 0.19)
Fruit	Reference	0.02 (−0.23 to 0.27)	−0.11 (−0.51 to 0.29)
Cooked or canned beans	Reference	0.16 (0.09 to 0.23)	0.18 (0.06 to 0.29)
Dark green vegetables	Reference	−0.05 (−0.12 to 0.02)	−0.08 (−0.20 to 0.04)
Orange-colored vegetables	Reference	0.14 (0.07 to 0.21)	0.05 (−0.06 to 0.17)
Other vegetables	Reference	0.00 (−0.76 to 0.08)	−0.16 (−0.29 to −0.03)

a Values are reported as times per day items were consumed. Differences were examined with analysis of covariance with Bonferroni post hoc tests. Analyses were adjusted for age; pregnancy, breastfeeding, and food security status; educational attainment; and social desirability trait. Intake of other vegetables was higher among non-Hispanic white or other participants than among non-Hispanic blacks (adjusted mean difference = 0.16, 95% CI [0.02–0.30]).

b Values were obtained by subtracting the mean frequency of intake of each group from the mean frequency of intake among Hispanics (reference group).

Among Hispanic participants, we found a significant association between origin and intake of other vegetables (Table 3). Columbian participants more frequently ate vegetables in the “other vegetables” category than Dominican participants (adjusted mean difference, 0.25; 95% CI, –0.47 to –0.03; Cohen *d* = 0.71; *P* = .01), and participants of other Hispanic origin more frequently ate other vegetables than did Dominican participants (adjusted mean difference, 0.19; 95% CI, –0.34 to –0.03; Cohen *d* = 0.52; *P* = .008). A significant association was found between nativity and eating orange-colored vegetables. Intake of orange-colored vegetables was higher among foreign-born than US-born participants (adjusted mean difference, 0.11; 95% CI, –0.20 to –0.02; Cohen *d* = 0.31; *P* = .02) (Table 4). Fruit and vegetable intake did not differ by language preference among foreign-born Hispanics.

**Table 3:** Differences in Intake of Fruits and Vegetables by Origin Among Hispanic Women in the Special Supplemental Nutrition Program for Women, Infants, and Children, 2015^a^^,^^b^

Food	Adjusted Mean Difference (95% Confidence Interval)					
	Dominican (N = 159)	Puerto Rican (N = 103)	Mexican (N = 43)	Columbian (N = 28)	Peruvian (N = 33)	Other Hispanic (N = 70)
100% fruit juice	Reference	−0.01 (−0.27 to 0.25)	0.07 (−0.25 to 0.27)	0.02 (−0.32 to 0.36)	0.11 (−0.21 to 0.43)	0.21 (−0.22 to 0.26)
Fruit	Reference	0.24 (−0.31 to 0.79)	−0.05 (−0.72 to 0.62)	0.42 (−0.30 to 1.13)	0.43 (−0.25 to 1.12)	−0.10 (−0.61 to 0.42)
Cooked/canned beans	Reference	0.06 (−0.09 to 0.22)	0.06 (−0.13 to 0.24)	0.21 (−0.01 to 0.40)	0.19 (−0.00 to 0.39)	0.10 (−0.05 to 0.24)
Dark green vegetables	Reference	0.09 (−0.09 to 0.27)	−0.05 (−0.27 to 0.17)	0.05 (−0.18 0.29)	0.10 (−0.12 to 0.33)	−0.04 (−0.21 to 0.13)
Orange-colored vegetables	Reference	0.07 (0.10 to 0.24)	−0.04 (−0.24 to 0.16)	−0.01 (−0.23 to 0.20)	−0.04 (−0.25 to 0.16)	−0.01 (−0.16 to 0.15)
Other vegetables	Reference	−0.13 (−0.31 to 0.04)	−0.06 (−0.27 to 0.15)	−0.25 (−0.47 to −0.03)	−0.01 (−0.23 to 0.20)	−0.19 (−0.34 to −0.03)

a Values are reported as times per day items were consumed. Differences were examined with analysis of covariance with Bonferroni post hoc tests. Analyses were adjusted for age; pregnancy, breastfeeding, and food security status; educational attainment; and social desirability trait.

b Values were obtained by subtracting the mean frequency of intake of each group from the mean frequency of intake among Dominicans (reference group).

**Table 4:** Differences in Intake of Fruits and Vegetables by Nativity Among Hispanic Women in the Special Supplemental Nutrition Program for Women, Infants, and Children, 2015^a^^,^^b^

Food	Adjusted Mean Difference (95% Confidence Interval)	
	US-born, n = 192	Foreign-born, n = 244
100% fruit juice	Reference	−0.08 (−0.22 to 0.06)
Fruit	Reference	−0.03 (−0.33 to 0.27)
Cooked or canned beans	Reference	−0.01 (−0.09 to 0.07)
Dark green vegetables	Reference	0.02 (−0.08 to 0.11)
Orange-colored vegetables	Reference	−0.11 (−0.20 to −0.02)
Other vegetables	Reference	−0.07 (−0.17 to 0.02)

a Values are reported as times per day items were consumed. Differences were examined with analysis of covariance with Bonferroni post hoc tests. Analyses were adjusted for age; pregnancy, breastfeeding and food security status; educational attainment; and social desirability trait.

b Values were obtained by subtracting the mean frequency of intake of each group from the mean frequency of intake among US-born participants (reference group).

## Discussion

We found differences by race/ethnicity in intake of cooked or canned beans, orange-colored vegetables, and vegetables in the other vegetables category. Among Hispanics, we found differences by origin in intakes of other vegetables and by nativity in intake of orange-colored vegetables. Language preference was unrelated to fruit and vegetable intake among foreign-born Hispanics, and intake of fruit and fruit juice did not differ by any of the demographic variables studied. The magnitude of differences in times per day vegetables were consumed ranged from 0.31 to 0.71.

Differences by race/ethnicity in the frequency of eating cooked or canned beans were not surprising in light of previous findings, thereby confirming existing knowledge of acculturation, cultural practices, and food preferences (3). However, other differences between our findings and previous findings were evident. Whereas Hispanics consumed more fruit, noncitrus fruit, and fruit juice than non-Hispanic blacks and non-Hispanic whites in other studies (4,5), intake of fruit and 100% juice did not differ by race/ethnicity in our study, possibly because of the low-income population that made up the sample. One study of low-income adults found that fruit and juice intake did not differ by race/ethnicity (21). A qualitative study conducted among low-income Hispanic women showed that food choices were shaped by cultural food preferences; a meal of rice, beans, and meat was considered healthy, satisfying, and more affordable than a meal including such healthy items as fruit and fruit juice (22). Such preferences may explain why Hispanic participants in our study did not consume fruit and 100% fruit juice more often than non-Hispanic blacks or non-Hispanic whites.

Why Hispanics ate orange-colored vegetables more often than non-Hispanic black participants is unclear. Previous work showed that neighborhood availability of orange-colored vegetables was associated with consumption of these foods and that convenience and corner stores in Hispanic neighborhoods were more likely to carry items such as carrots than stores in black neighborhoods (23,24). Alternatively, findings may be explained by differences between racial/ethnic groups in preferences for orange-colored vegetables such as carrots and winter squash. A study of carotenoid intake and profiles found that non-Hispanic whites obtained most α-carotene and β-carotene from carrots and mixed vegetables. Although these vegetables also were sources among Hispanics, winter squash (frequently included in the preparation of beans) was a more significant source (3). Moreover, although non-Hispanic whites obtained important amounts of lutein and zeaxanthin from leafy green vegetables (spinach and broccoli), Hispanics obtained more of these nutrients from winter squash (3).

Also unclear is why the frequency of intake of other vegetables was higher among non-Hispanic white participants than non-Hispanic blacks or Hispanics. A study of adults found that non-Hispanic whites consumed more other vegetables than Hispanics (5); however, the sample did not include non-Hispanic blacks. Potatoes are counted among other vegetables assessed by the BRFSS fruit and vegetable module (18). Previous work showed that non-Hispanic white women eat potatoes more often than Hispanic women, which may explain the higher intake of other vegetables found in this group (3).

Among Hispanics, Columbians had a higher frequency of intake of other vegetables than did Dominican and other Hispanic participants. Colón-Ramos et al similarly found that South American women (including Columbians) had higher intakes of other white potatoes (potatoes other than French fries, home fries, or hash browns, an item comparable to non-fried potatoes counted among other vegetables by the BRFSS fruit and vegetable module) than did Caribbean women (including Dominicans) and higher intakes of lettuce (also counted among other vegetables by the BRFSS) than did Central American women (including Salvadorians, Guatemalans, Costa Ricans, Hondurans, Nicaraguans, and Panamanians) (9). Yet, our findings are not directly comparable to the findings of Colón-Ramos et al because multiple origins were subsumed within the Hispanic groups studied by Colón-Ramos et al. In other work, total vegetable intakes differed among Cubans, Dominicans, and Puerto Ricans, and intakes of dark green/yellow and non-starchy vegetables differed between Puerto Rican and non-Puerto Rican Hispanic women (10). Together with previous findings (25), our findings suggest that vegetable intakes differ by Hispanic origin. The few studies to date underscore the need for further research of this type. Studies of vegetable intakes in diverse Hispanic origin groups may aid understanding of similarities and differences between groups.

Most foreign-born Hispanics preferred to speak Spanish, suggesting that they were less acculturated than foreign-born Hispanics who preferred to speak English. The higher intake of orange-colored vegetables among foreign-born than US-born Hispanics is consistent with literature demonstrating an inverse association between acculturation and fruit and vegetable intake (7,8). The homogeneity of language preference among foreign-born Hispanics may also explain why fruit and vegetable intake did not differ by language preference in this group.

Our study had limitations. Fruit and vegetable intake was assessed by self-report, which may lead to misclassification and bias (9). To minimize bias, analyses were controlled for social desirability trait, a factor associated with overestimation of self-reported intake (26). The self-selected sample and possible self-selection bias resulting from the high refusal rate limit the generalizability of findings. Findings may be generalizable only to WIC populations with characteristics similar to those in our sample (25). Findings of this study are not directly comparable to findings of previous studies because of the few similar studies conducted among low-income women in general and among women in WIC in particular. Most Hispanic participants in this study were Dominican or Puerto Rican. Intake patterns observed among Hispanics may therefore better reflect patterns found among Dominicans and Puerto Ricans than among Hispanics in general. Although this study was powered to detect moderate to large effects, observed effect sizes were small to medium by Cohen’s conventions (20). As such, there was insufficient power for some pairwise comparisons, highlighting the need for replication studies with sufficiently large subgroups of race/ethnicity, origin, and nativity.

Despite these limitations, our findings add to the limited data on fruit and vegetable intake among low-income women in the WIC program. Findings advance understanding of variations in intake based on race/ethnicity and Hispanic origin and nativity. Fruit and vegetable intake was assessed with a validated measure in widespread use thereby facilitating comparisons with intake estimates derived from other similar studies reporting intake as times per day fruit and vegetables were consumed. To our knowledge, this is the first study to examine fruit and vegetable intake among the Hispanic origin groups we included. Most research to date has focused on Mexican Americans (9); therefore, our findings extend knowledge of intake differences among these groups. Analyses were controlled for several potential confounders, among them, social desirability trait. Although shown to bias self-reported dietary intake, social desirability assessments have not been included in previous studies of this type.

Although reasons for the differences found in fruit and vegetable intake were considered, further research is needed to confirm factors that explain the differences. In addition to country of origin, future studies should collect data on geographic region of origin, because ingredients, preparation techniques, the use of culinary marinades and seasonings, and the names of similar foods and dishes can vary by country and from one region to another within the same country (27). This study was conducted in the summer. Fruit and vegetable availability and consumption vary seasonally (28). Such variations may have affected the results. The collection of dietary data at different times of the year is therefore recommended in future studies. BRFSS fruit and vegetable items encompass several foods. To better understand group differences in item intakes, assessments of foods counted in the definition of each are needed. We also recommend assessments of the neighborhood availability and accessibility of commonly consumed and culturally specific items because limited availability and accessibility may serve as barriers to consumption.

Among WIC participants, vegetable intake differed by race/ethnicity, and among Hispanics, by origin and nativity. Differences found for some fruits and vegetables but not others underscore the need for WIC nutrition policies to focus on fruit and vegetable components (particularly those for which intakes are furthest from meeting recommendations) rather than fruit and vegetable intake overall. The success of WIC fruit and vegetable interventions may also be enhanced by adapting messages and materials to the needs of participants who differ by race/ethnicity and among Hispanics, by origin and nativity. Replication studies with large and diverse Hispanic origin groups are needed to confirm study findings. To aid understanding of components of dietary intake, the use of dietary assessment methods that allow for the collection of detailed information on food and juice intakes (eg, 24-hour recalls) is recommended. Attention to geographic region of origin in addition to country of origin also may advance understanding of differences in fruit and vegetable intake within Hispanic origin groups.

## Appendix. Foods Assessed by the Behavioral Risk Factor Surveillance System Fruit and Vegetable Module and Corresponding Foods and Juices Included and Excluded in the Definition of Each

This file is available for download as a Microsoft Word document.
