# Supplementary material for: Differences in Fruit and Vegetable Intake by Race/Ethnicity and by Hispanic Origin and Nativity Among Women in the Special Supplemental Nutrition Program for Women, Infants, and Children, 2015
Source: Prev Chronic Dis. 2016 Aug 25;13:E115. doi: 10.5888/pcd13.160130 (PMC5003529; doi:10.5888/pcd13.160130)
Supplement: Supplementary file 1 [file 16_0130_Appendix.docx]

**Appendix. Foods Assessed by the Behavioral Risk Factor Surveillance System Fruit and Vegetable Module and Corresponding Foods and Juices Included and Excluded in the Definition of Each**

| **Food** | **Included** | **Excluded** |
| --- | --- | --- |
| 100% fruit juice | • 100% pure juices including orange, mango, papaya, pineapple, apple, grape (white or red), or grapefruit  • Cranberry juice if the respondent’s perception is that it is 100% juice with no sugar or artificial sweetener added  • 100% juice blends such as orange-pineapple, orange-tangerine, and cranberry-grape and fruit-vegetable 100% blends  • 100% pure juice from concentrate (ie, reconstituted) | • Fruit flavored drinks with added sugar  • Fruit juice made at home that sugar was added to  • Fruit drinks with added sugar or other added sweeteners like Kool-Aid, Hi-C, lemonade, cranberry cocktail, Tampico, Sunny Delight, Snapple, Fruitopia, Gatorade, Power-Ade or yogurt drinks  • Fruit juice drinks that provide 100% daily vitamin C but include added sugar  • Fruit jam, jelly, or fruit preserves  • Vegetable juices such as tomato and V-8 |
| Fruit | • Fresh, frozen, or canned fruit  • Apples, bananas, applesauce, oranges, grapefruit, fruit salad, watermelon, cantaloupe or musk melon, papaya, lychees, star fruit, pomegranates, mangos, grapes, and berries such as blueberries and strawberries  • Dried raisins and cran-raisins  • Cut up fresh, frozen, or canned fruit added to yogurt, cereal, jello, and other meal items  • Culturally and geographically appropriate fruits, eg, genip, soursop, sugar apple, figs, tamarind, bread fruit, sea grapes, carambola, longans, lychees, akee, rambutan, etc. | • Fruit jam, jelly or fruit preserves  • Dried fruit in ready-to-eat cereals |
| Cooked or canned beans | • Refried, baked, black, garbanzo beans, beans in soup, soybeans, edamame, tofu or lentils including round or oval beans or peas such as navy, pinto, split peas, cow peas, hummus, lentils, soybeans (also called edamame) and tofu (bean curd made from soybeans), kidney, pinto, hummus, lentils, black, black-eyed peas, cow peas, lima beans and white beans  • Bean burgers including garden burgers and veggie burgers  • Falafel and tempeh | • Long green beans such as string beans, broad or winged beans or pole beans |
| Dark green vegetables | • Broccoli or dark leafy greens including romaine, chard, collard greens or spinach  • All raw leafy green salads including spinach, mesclun, romaine lettuce, bok choy, dark green leafy lettuce, dandelions, komatsuna, watercress and arugula  • All cooked greens including kale, collard greens, choys, turnip greens and mustard greens | • Iceberg (head) lettuce |
| Orange-colored vegetables | • Sweet potatoes (baked, mashed, casserole, pie, or sweet potato fries), pumpkin (including pumpkin soup and pie), winter squash (squash with hard, thick skins and deep yellow to orange flesh including acorn, buttercup, spaghetti squash, autumn cup, banana, butternut, delicate, hubbard and kabocha [also known as an Ebisu, Delica, Hoka, Hokkaido, or Japanese Pumpkin; blue kuri] and all forms including soup) and carrots (all forms including long or baby-cut and carrot-slaw [e.g. shredded carrots with or without other vegetables or fruit]) | • Pumpkin bars, cake, bread or other grain-based desert-type food containing pumpkin similar to banana bars and zucchini bars |
| Other vegetables | • Tomatoes, tomato juice or V-8 juice, corn, eggplant, peas, lettuce, cabbage (all cabbage including American-style cole-slaw), white potatoes that are not fried such as baked or mashed potatoes, okra, beets, cauliflower, bean sprouts, avocado, cucumber, onions, peppers (red, green, yellow, orange), mushrooms, snow peas, snap peas, broad beans and string, wax-, or pole-beans  • Raw, cooked, canned, or frozen vegetables  • Culturally and geographically appropriate vegetables, e.g., daikon, jicama, oriental cucumber, etc. | • Fried potatoes  • Rice or other grains  • Products consumed usually as condiments including ketchup, catsup, salsa, chutney and relish |
